# A microRNA Encoded by Kaposi Sarcoma-Associated Herpesvirus Promotes B-Cell Expansion *In Vivo*

**DOI:** 10.1371/journal.pone.0049435

**Published:** 2012-11-20

**Authors:** Christine Dahlke, Katrin Maul, Thomas Christalla, Nicole Walz, Philipp Schult, Carol Stocking, Adam Grundhoff

**Affiliations:** Heinrich-Pette-Institute, Leibniz Institute for Experimental Virology, Hamburg, Germany; Lisbon University, Portugal

## Abstract

The human gammaherpesvirus Kaposi sarcoma-associated herpesvirus is strongly linked to neoplasms of endothelial and B-cell origin. The majority of tumor cells in these malignancies are latently infected, and latency genes are consequently thought to play a critical role in virus-induced tumorigenesis. One such factor is kshv-miR-K12-11, a viral microRNA that is constitutively expressed in cell lines derived from KSHV-associated tumors, and that shares perfect homology of its seed sequence with the cellular miR-155. Since miR-155 is overexpressed in a number of human tumors, it is conceivable that mimicry of miR-155 by miR-K12-11 may contribute to cellular transformation in KSHV-associated disease. Here, we have performed a side-by-side study of phenotypic alterations associated with constitutive expression of either human miR-155 or viral miR-K12-11 in bone marrow-derived hematopoietic stem cells. We demonstrate that retroviral-mediated gene transfer and hematopoietic progenitor cell transplantation into C57BL/6 mice leads to increased B-cell fractions in lymphoid organs, as well as to enhanced germinal center formation in both microRNA-expressing mouse cohorts. We furthermore identify Jarid2, a component of Polycomb repressive complex 2, as a novel validated target of miR-K12-11, and confirm its downregulation in miR-K12-11 as well as miR-155 expressing bone marrow cells. Our findings confirm and extend previous observations made in other mouse models, and underscore the notion that miR-K12-11 may have arisen to mimic miR-155 functions in KSHV-infected B-cells. The expression of miR-K12-11 may represent one mechanism by which KSHV presumably aims to reprogram naïve B-cells towards supporting long-term latency, which at the same time is likely to pre-dispose infected lymphocytes to malignant transformation.

## Introduction

Kaposi sarcoma-associated herpesvirus (KSHV) is the etiological agent of Kaposi's sarcoma (KS), a tumor of endothelial origin, as well as the lymphoproliferative disorders primary effusion lymphoma (PEL) and multicentric Castleman's disease (MCD) [1]–[3]. Like all herpesviruses, KSHV can establish long-term latent infections of its host cell. The natural target cell of KSHV infection *in vivo* is the B-cell, and latently infected memory B-cells are thought to represent a reservoir that is of importance for the lifelong KSHV persistence. Latently infected cells do not produce viral progeny; rather, the virus persists as a nuclear replicating episome that expresses only a small repertoire of its genes. It is thought that these latency genes primarily serve to uphold latent infection and ensure survival of the host cell. However, as the majority of cells in KSHV-associated malignancies are latently infected, it is also likely that latent gene products play a pivotal role during virally induced tumorigenesis. Latent KSHV genomes minimally express three protein-coding genes, all of which are transcribed from a single multicistronic locus. These transcripts express the multifunctional latency-associated nuclear antigen LANA, a viral cyclin D homologue (v-Cyc) and a viral homologue of a FLICE-inhibitory protein (v-Flip) [4]–[9]. Furthermore, we and others have recently shown that primary latency transcripts also produce at least 25 mature microRNAs (miRNAs) that are encoded by a total of 12 microRNA precursor (pre-miRNA) genes [10]–[14].

miRNAs are small, non-coding RNAs with a length of ∼22 nucleotides (nt) that are encoded by virtually all multicellular organisms and some viruses. miRNAs bind to the 3′UTR of target mRNA transcripts with partial or perfect sequence complementarity, resulting in translational repression and/or mRNA destabilization [15]. Although the precise parameters that govern target recognition remain incompletely understood, the great majority of functional target sites minimally exhibit perfect Watson-Crick pairing with the so-called seed region (nts. 2–7) of the miRNA [16]. While seed complementarity is a necessary and sufficient requirement for the functionality of most target sites, auxiliary base pairing at the 3′-end of the miRNA can augment the efficacy of some sites, and a subset of non-canonical target sites may be recognized in the absence of perfect seed pairing [16], [17]. miRNA target transcripts are thought to form complex networks that co-evolve with a given miRNA, and that may can contain hundreds of members [16], [18]. However, in the majority of cases, the precise contribution of individual targets the function of a given miRNA remains unknown.

More than 90% of the viral miRNAs that have been identified to date are encoded in the genomes of herpesvirus family members, suggesting that usage of such molecules may be especially beneficial for viruses that establish long-lasting infections. In accord with such a notion, a number of studies have suggested that miRNAs support latent infection by controlling expression of viral as well as host genes (see [19]–[21] and references therein). Interestingly, although herpesvirus miRNAs show little evolutionary conservation beyond the species level [20], [22], [23], recent large scale studies have nonetheless found that diverse viral miRNAs may regulate common sets of target transcripts, arguing for functional conservation despite sequence variability [24]. While most viral miRNAs have unique seeds, a minority shares their seed sequence with host miRNAs and thus may allow the virus to gain access to pre-existing networks of host target transcripts. In this regard, the KSHV-encoded miR-K12-11 is an especially interesting case: miR-K12-11 shares its seed with the cellular miR-155, a miRNA that plays important roles during hematopoiesis and the regulation of germinal center responses [25]–[27]. Since miR-155 expression is furthermore frequently deregulated in lymphoma, leukemia and breast cancer [28]–[31], and since forced miR-155 expression has been found to induce proliferative disease in mice [28], [32], it has been proposed that mimicry of miR-155 functions by kshv-miR-K12-11 may contribute to the development of KSHV-associated tumors [21], [24]. Indeed, several studies have found that kshv-miR-K12-11 and miR-155 regulate an overlapping set of target genes [19], [21], [24]. Further support for this hypothesis comes from the observation that the oncogenic Marek's disease virus type 1 (MDV-1), an alphaherpesvirus that induces T-cell lymphoma in birds, also encodes a miRNA with an identical seed as miR-155 and loses its oncogenic potential in vivo when the miRNA is mutated [33], [34]. Additionally, Epstein-Barr Virus (EBV) and reticuloendotheliosis virus strain T (REV-T), two oncogenic viruses that do not encode their own miR-155 mimics, induce expression of the host miR-155 during infection [35]–[39]

To investigate to what extend kshv-miR-K12-11 may mimic miR-155 functions *in vivo*, we have explored phenotypic consequences of seed sharing by constitutively expressing physiological levels of each miRNA in the hematopoietic system of C57BL/6 mice, using retroviral transduction and hematopoietic stem cell transplantation technology. Although a murine background would not be suitable to study heterologous viral miRNAs that do not represent analogs of conserved cellular miRNAs, we reasoned that such a system would offer distinct advantages to investigate a suspected host miRNA mimic: If kshv-miR-K12-11 has indeed evolved to phenocopy miR-155, it should also retain its ability to target evolutionary conserved miR-155 target sites and thus remain fully functional in murine cells. Indeed, we find that both miRNAs mediate expansion of CD19^+^ B-cells in the spleen, as well as an increase in the frequency of CD19^+^/CD43^−^ pre-B-cells in the bone marrow (BM). Furthermore, we observe higher numbers of germinal centers (GCs) and increased GC areas in miRNA expressing mice, indicating that one benefit of expressing kshv-miR-K12-11 may be to allow infected B-cells to transit GCs and gain access to the memory B-cell compartment. Additionally, we show downregulation of known miR-155 and/or kshv-miR-K12-11 targets in miRNA-expressing BM cells *in vivo* and confirm Jarid2 as a novel target of kshv-miR-K12-11. Jarid2, the founding member of the Jumonji C (JmjC)-domain protein family, regulates the activity of polycomb repressor complexes (for reviews, see [40], [41]). Jarid2 plays an important role during embryonic development and cell differentiation, functions as a tumor suppressor in hematological disorders, and was shown to suppress cell proliferation as well as to negatively influence B-cell survival. Taken together, our data thus support previous notions of kshv-miR-K12-11 having evolved to mimic miR-155 functions, confirm observations made in other mouse models of forced kshv-miR-K12-11/miR-155 expression [42], and suggest that Jarid2 is one of the factors that is targeted by kshv-miR-K12-11 during viral infection as well as in KSHV-associated disease.

## Materials and Methods

### Ethics Statement

Animal experiments were handled in strict accordance with good animal practice and national and international guidelines for care and use of experimental animals. All animal experiments and protocols were approved by the Hamburg Office of Health (permit no. Fl87/04 and 79/08). All efforts were made to minimize animal suffering

### Plasmid constructs

Genomic fragments of ∼350 bp encoding pre-miRNA sequences for kshv-miR-K12-11, kshv-miR-K12-7 and hsa-miR-155 were amplified from BCBL-1 and Raji DNA using the following primers: K12-11fw ATC GGA TCC ATC TAG TCG C CC GTT ATT GT; K12-11rev GAT GAA TTC CAC GCG TAC GGT GGT CTC AT, K12-7fw ATC GGA TCC GCA ATT TTT GTC GTA TGC GC; K12-7rev GAT GAA TTC GGA TAG CCA CCC ACA ATT GT, miR-155fw AGG GAT CCA CTA TAT GCT GTC ACT CCA GCT; miR-155rev, CAA GAA TTC CCA GTG ACC AGA TTA TGA TTA AC. The pre-miRNA sequences were inserted into EcoRI and BamHI restriction sites downstream from the promoter and GFP gene of the retroviral vector RSF91 [43]. A Jarid2 expression vector was obtained from ATCC (ATCC number 9121591, IMAGE Clone 4520786, GenBank accession BC046246).For luciferase reporter assays, the full-length Jarid2 3′ UTR was amplified and inserted into SpeI and HindIII restriction sites of the pMIR-report (Ambion) vector using the following primers: Jarid2fw_SpeI, AGT ACT AGT GCC CGT GGT CGA TTT ATA; Jarid2rev_HindIII, GGC GAA GCT TTG AAG TCT CCC TCC AAG). Constructs harbouring mutations in seed match regions 1 and 2 were generated in using the Quikchange site-directed mutagenesis protocol (Stratagene), using pMIR-Jarid2 as backbone vector. The primer sequences used to introduce mutation at binding sites 1 and 2 were: Jarid2mut1fw, GAG AAC TAA TTT TGT TTT AGG CAT AAA CTG TTG; Jarid2mut1rev, CAA CAG TTT ATG CCT AAA ACA AAA TTA GTT CTC; Jarid2mut2fw, GAC ACC TTC ACA AGT CAG GCA TAA TCT TTC; Jarid2mut2rev, GAA AGA TTA TGC CTG ACT TGT GAA GGT GTC. The sequences of all inserts were confirmed by Sanger sequencing.

### Mice

C57BL/6 mice were bred in the Heinrich-Pette-Institute animal quarters under SPF conditions.

### Cell lines and cell culture

293T (ATTC cat. number CRL-11268) and NIH3T3 (ATTC cat. number CRL-1658) cells were both cultured in Dulbecco's modified Eagle's medium (DMEM) supplemented with 10% fetal calf serum (FCS) and 1% Penicillin-Streptomycin with 5% CO_2_ at 37°C. Bone marrow cells (BM) isolated from wild type (wt) C57BL/6 mice were cultured in StemSpan Serum-free expansion medium (StemCell Technologies)) supplemented with 1% glutamine, 1% penicillin-streptomycin, 100 ng/mL each murine stem cell factor (Peprotech), human Flt3-ligand and human interleukin 11 (Peprotech), and 10 ng/mL murine interleukin 3 (Peprotech). The KSHV-positive PEL cell line BCBL-1 [44], the KSHV-negative/EBV-positive Burkitt's lymphoma (BL) cell line Raji (ATTC cat. number CCL-86), and the EBV-positive lymphoblastoid cell line (LCL) LCL 721 [45] were carried in RPMI 1640 supplemented with 10% FCS and 1% Pen/Strep at 37°C with 5% CO2. Suspension cells were grown to a density of 1–2× 10E6 cells/ml and then split 1∶5 in fresh medium.

### Transfection

Transient transfection of 293T cells (5×10^6^ cells for 10 cm cell dish or 4×10^5^ cells for 6-well dish) was performed using a 1 mg/ml polyethyleneimine (PEI, Sigma-Aldrich, Taufkirchen) solution. Cell culture medium was removed and cells were overlaid with OptiMem (Invitrogen). Then, 1–5 µg DNA was diluted in 1 ml OptiMem and mixed with 1 mg/ml PEI. The mixture was incubated for 15 min at RT and subsequently added drop by drop to the cells in medium. The cells were incubated for 8 h at 37°C. Then the supernatant was removed and the transfected cells were overlaid with fresh medium with supplements.

### Retroviral gene transduction and hematopietic stem cell/progenitor cell transplantation

Retroviral vector pseudotypes were generated by transient transfection of 293T cells (5×10^6^ cells in a 10 cm cell dish) using PEI plus plasmids expressing viral packaging proteins (ecotropic Env; 3 µg of pEcoenv-I-puro [46] and gag-pol (8 µg of pSV40-gag-pol [47]) and the retroviral vector (5 µg). After 48 h, 72 h and 96 h the supernatant was harvested, sterile filtered through 0.22 µM Millex-GV Filter (Millipore) and used for transduction of hematopietic progenitor cells/stem cells (HPC/HSC) isolated from bone marrow or of NIH3T3 cells. Virus titer was determined using GFP-expression analysis via flow cytometry (FACS Canto; BD Bioscience) three days post transduction of NIH3T3 cells.

For the transduction of NIH3T3 cells, 5×10^4^ cells were seeded into a 24-well plate and infected by spinoculation (28°C, 500×g, 1 h) using ∼1×10^6^ infectious particles per mL of titered stocks supplemented with FCS and polybrene (8 µg/µL; Sigma-Aldrich, Taufkirchen) Virus suspensions with a titer of ∼1×10^6^ infectious particles per mL were used for transductions of primary BM cells. To enrich for hematopoietic progenitors, BM cells were harvested from tibiae and femora of donor wt C57BL/6 mice that received an intraperitoneal injection of 5-fluorouracil (150 mg/kg; GRY-Pharma GmbH) five days prior to isolation; or BM cells were harvested from untreated C57BL/6 mice and subject to negative lineage depletion using a Lineage Depletion Kit (Miltenyi Biotec). For hematopoietic progenitor transductions, virus particles were fixed onto non-tissue 35-mm culture plates coated with Retronectin (Cambrex) by centrifugation for 4×30 min at 400×g at 4°C. HPC/HSC were suspended in StemSpan SFEM medium (StemCell Technologies) with supplements (see cell culture) before addition to virus-enriched cultures. Four rounds of transduction were performed.

Transduced HPC/HSC (1×10^6^ cells) supplemented with untreated splenic cells (5×10^4^) were transplanted into lethally irradiated (9 Gy) C57BL/6 mice by tail vein injection. After 12 to 16 weeks, the mice were sacrificed and hematopoietic organs were analyzed. Three independent BM transductions and transplantations were performed.

### Flow cytometry analysis

Single cell suspensions from spleens were prepared by pressing through a 100 µm microstrainer. Erythrocytes were depleted using Erylysis buffer (Pharm Lyse; BD Biosciences). Splenocytes and BM cells were stained with following conjugated antibodies for 1 h in PBS supplemented with 1% FCS: anti-CD19-PE (clone 1D3, Pharmingen), anti-CD19-APC (clone 6D5, BioLegend), anti-B220-APC (clone RA3-6B2, BioLegend), anti-CD3e-PE (clone 145-2C11, BioLegend), anti-CD11b-PE (clone M1/70, BioLegend, anti-Gr1-APC (clone RB6-8C5, eBioscience). Flow cytometry analysis and cell sort of GFP positive cells (BM cells, 293T, NIH3T3) were performed on a FACSAriaII (BD Bioscience). Data were analyzed using FACSDiva software version 6.1.3.

### miRNA Expression: Real-time stem-loop RT PCR

To compare miRNA expression levels of transfectants (293T) and transcductants (NIH3T3) with the positive controls BCBL-1 or RAJI, we extracted total RNA of GFP^+^ flow sorted cells as well as from BCBL-1 and RAJI using RNA Bee (AMS Biotechnology, Milton, UK). Reverse transcription (RT) was performed using Superscript III (Invitrogen). For each sample the same input of total RNA (1 µg) was added to the RT mix together with the miRNA specific stem-loop primers for hsa-miR-155 (miR-155_SL GTT GGC TCT GGT GCA GGG TCC GAG GTA TTC GCA) and kshv-miR-K12-11 (miR-K12-11_SL GTT GGC TCT GGT GCA GGG TCC GAG GTA TTC GCA). The cDNA synthesis was performed in accordance to the protocol from Varkonyi-Gasic [39]. For each Real-Time PCR reaction, 1.5 µl cDNA was added to the real-time PCR mix. Real-time PCR was performed on the Rotor Gene Q (Qiagen, Hilden Germany), using Rotor-Gene Multiplex PCR Kit (Qiagen) and the following forward pimers: kshv-miR-K12-11_fw, GCT GTT AAT GCT TAG CCT GT or hsa-miR-155_fw CAG CTT AAT GCT AAT CGT GAT, and the universal reverse primer UniRev, GTG CAG GGT CCG AGG T. The efficiency of both primer pairs was evaluated using a ten-fold serial dilutions of BCBL-1 and Raji cDNA, and the expression levels of kshv-miR-K12-11 and hsa-miR-155 in GFP^+^ flow sorted transductants and transfectants was normalized to BCBL-1 or Raji, respectively. Runs were analyzed via Rotor Gene software, and each experiment was performed using three biological replicates, in which each sample was analyzed in duplicate.

### mRNA Expression: Real-time PCR

Total RNA derived from GFP^+^ flow sorted BM cells was extracted using RNA Bee (AMS Biotechnology, Milton, UK) per the manufacturer's instructions. cDNA synthesis was performed using Superscript III (Invitrogen) per manufacturer's instructions in the presence of random hexamers. 15 ng of total RNA was added to the RT mix. Real-time PCR was performed on the Rotor Gene Q with 1.5 µl of cDNA and SensiMix SybrGreen (peqlab), using the following primers: Jarid2fw, 5′- CCC AAG TGT CCT CCA CTA GC -3′; Jarid2fw, 5′- TGG GAC TAT TCG GCT GAG AC -3′. mRNA levels were normalized using the housekeeping genes mmu RPLP (fw 5′- CTC GCT TGC ATC TAC TCC GC, rev 5′- AGA AAG GTT CGA CGC TGA CAC -3′), mmu Actin (5′- GAA ATC GTG CGT GAC ATC AAA G, rev 5′- TGT AGT TTC ATG GAT GCC ACA G -3′) and mmu GAPDH (fw 5′- GGT GAA GGT CGG TGT GAA C -3′, rev 5′- GGG GTC TCG CTC CTG GAA -3′). Calculation of normalized expression levels was performed according to Vandesompele et al. [48], using the geNorm calculation scheme available at http://medgen.ugent.be/~jvdesomp/genorm/example_calculations.xls.

### Luciferase reporter assay

miRNA activity was analyzed using the luciferase assay system pMIR-report™. 293T cells (2×10^4^) were seeded in each well of a 96 well plate. After 24 h cells were transfected with the pMIR-report construct (50 ng), the pcDNA3-GFP-miRNA construct (50 ng) and a ß-galactosidase expressing vector (50 ng) for normalization. Transfections were done in triplicate using three independent rounds of transfection, which were performed with PEI (1 µl per reaction) in OptiMem (25 µl per reaction; Invitrogen) medium. Lysates were prepared 24 h post transfection for the measurement of luciferase and ß-galactosidase activity. Cells were washed once in 50 µl PBS and then lysed in 100 µl 1× RLB (Renilla Lysis Buffer) using one freeze/thaw cycle. Prior measurement, lysates were homogenized by pipetting. The galactosidase activity was measured by transferring 50 µl lysate in a new 96 well plate and mixing with 50 µl 2× Z-buffer plus o-nitrophenyl-ß-D-galactopyranoside (ONPG) and freshly added ß-mercaptoethanol (7 µl/ml). Once a faint yellow color developed, the reaction was stopped by adding 150 µl of 1 M NaCO3 and absorption at 420 nm was measured using a microplate reader (Synergy Mx) and the Gen5 data analysis software. Luminescence was measured using the Promega's Luciferase Assay System according to the manufacturer's instructions. The obtained light units were normalized to ß-galactosidase activity.

### Western blot analysis

Whole-cell lysates were harvested 48 h post transfection. Lysates, corresponding to about 5×10^5^ transfected 293T cells (protocol for transfection: see section above), were separated on standard SDS-PAGE (10%-gels) and transferred onto PVDF-membranes (Millipore). Blots were probed using antibodies directed against Jarid2 (Santa Cruz, sc-134548) or, as a loading control, actin (Santa Cruz, sc-47778).

### Histology and immunohistochemistry

Spleens were fixed in 4% neutral buffered formalin and embedded in paraffin. Deparaffind sections were stained according to standard laboratory protocols with hematoxylin and eosin staining (H&E). Sections were cut at 4 µm for H&E staining and for immunohistochemistry using PNA (Vector Laboratories, Burlingame, CA).

For PNA staining, the dewaxed slides were incubated with 1∶100 dilution of the primary antibody for 45 min. Detection was performed using ABC Elite Peroxidase Kit plus DAB-Kit (DAKO) and counterstained with Mayers hemalm. The samples were analyzed using a Zeiss microscope (Axioplan2 imaging) with the objective lens of 10x/0.45 Zeiss Plan-APOCHROMAT. Pictures were taken using Jenoptik ProgRes C12 and Imagic Jenoptik CxxPlus. The images were evaluated using Image Access Enterprise, Adobe Photoshop CS4 (Adobe, San Jose, CA) and Nikon PixelClassifier software (Nikon NIS elements). GC numbers and relative GC areas were determined across an entire splenic section from each of 15 GFP control, 18 kshv-miR-K12-11 and 12 hsa-miR-155 mice, using the Pixel Classifier software as per the manufacturer's instructions. The total number of GCs was determined by calculating the sum of GCs detected in all sections. To evaluate the relative GC area, areas of individual GCs were evaluated by marking the border of each GC and assigning it as region of interest (ROI), followed by automatic calculation of the total GC area and normalization to the total area of all splenic sections.

### Statistics

All statistical analyses were performed applying student's two-tailed t-test using GraphPad Prism 5.03 software.

## Results

### Generation of recombinant retroviruses for ectopic expression of kshv-miR-K12-11 and hsa-miR-155

**Figure 1:**
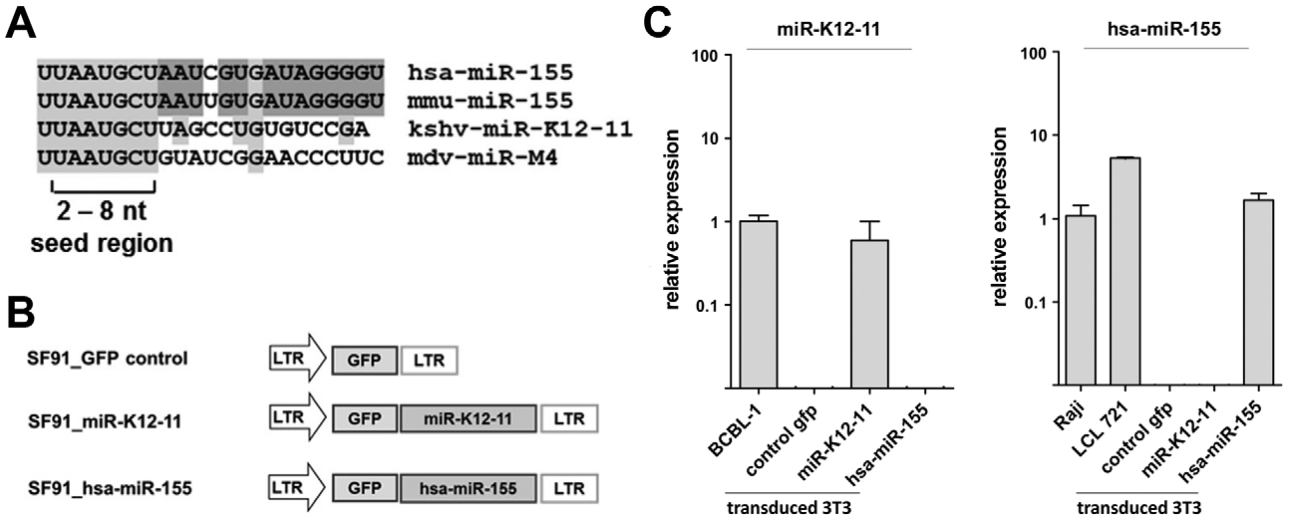
Retroviral constructs for ectopic miRNA expression. A) Seed sharing between viral and cellular miRNAs. Shown is an alignment of human (hsa) and murine (mmu) miR-155 orthologues, the KSHV-encoded miR-K12-11 and the MDV-encoded miR-M4. Nucleotides that are conserved between hsa- and mmu-miR-155 are shown on dark gray background, and nucleotides that are identical in two or more miRNAs are highlighted in light gray. The seed region (nts. 2–8) that is considered to be the most critical region for base-pairing between miRNA and target sites is marked. B) Schematic depiction of y-retroviral miRNA expression vectors. The precursor hairpins of kshv-miR-K12-11 and hsa-miR-155 are placed downstream of the GFP gene and transcribed from the LTR (long terminal repeat) promoter. C) Endogeneous and ectopic miRNA expression in transduced NIH 3T3 cells, and B-cell lymphoma lines, respectively. miRNA expression levels were analyzed by real-time stem-loop RT PCR in GFP^+^-sorted transduced NIH 3T3 cells or the indicated B cell lines. BCBL-1 and Raji were used as positive and normalization controls for kshv-miR-K12-11 or hsa-miR-155 expression, respectively, and relative expression levels in these cell lines was set to 1. All experiments were done in triplicate.

To study the effects of constitutive microRNA expression on the differentiation of hematopoietic progenitor cells we generated gamma-retroviral vectors that express GFP as well as the precursor hairpins (pre-miRNAs) of kshv-miR-K12-11 or hsa-miR-155 under the control of the viral promoter in the 5′ long terminal repeat (LTR) (Fig. 1B). A vector expressing GFP alone was used as a control. The basic hypothesis of our study was that the viral kshv-miR-K12-11 has evolved to mimic the host microRNA miR-155, thus the human orthologue of this microRNA (hsa-miR-155) was used as a positive control. As shown in Fig. 1A, the mature human microRNA differs from its murine counterpart (mmu-miR-155) in only a single nucleotide exchange at position 12 (see Fig. 1A). As this nucleotide is not involved in base pairings at canonical microRNA target sites [16], and since relevant microRNA target sites tend to be well conserved across species, we reasoned that hsa-miR-155 is very likely to remain fully functional in a murine background. Consequently, if the observed seed sharing is sufficient to allow kshv-miR-K12-11 to engage the functionally important contingent of host transcripts, similar phenotypes should ensue upon forced expression of the viral and human miRNAs in murine HSC.

To investigate the functionality of the generated constructs, we analyzed miRNA expression in GFP-positive NIH3T3 cells that had been transduced with recombinant virions. Expression levels were determined by real-time stem-loop RT-PCR [49] and normalized to the levels observed in the KSHV-positive PEL cell line BCBL-1 for miR-K12-11, or the KSHV-negative/EBV-positive Burkitt's lymphoma cell line Raji for hsa-miR-155. As an additional point of reference, we also analyzed hsa-miR-155 expression in an EBV-positive lymphoblastoid cell line (LCL 721), since such lines have previously been shown to express very high levels of this miRNA [36], [50]–[56]. All experiments were done in triplicate.

As shown in the left panel of Figure 1C, the viral miRNA was readily detectable in kshv-miR-K12-11 transduced NIH-3T3 cells, but not the GFP-only controls or cells that had received hsa-miR-155. The transduced 3T3 cells furthermore displayed expression levels that were slightly (1.3-fold) lower than those observed in BCBL-1 cells, indicating that transduction did not lead to overexpression of the viral miRNA. Based on a previous study that found approximately 1000 kshv-miR-K12-11 copies per cell of a BCBL-1 culture [42], we hence estimate that the transduced 3T3 cells express between 600 and 700 copies of the viral miRNA per cell. Our stem-loop PCR for hsa-miR-155 was also specific and revealed that transduced 3T3 cells expressed the cellular miRNA at levels that were only slightly higher as those seen in Raji (approximately 1.7-fold, see Figure 1C, right panel), but below those observed in LCL 721 cells. Although the observation of substantially (6.3-fold) higher hsa-miR-155 expression in LCL 721 relative to Raji cells was in general accord with previous observations of high level expression particularly in LCL cells [51]–[55], one study had reported a significantly larger difference between Raji and LCL cells (20- to 30-fold, [54]). Therefore, we sought to confirm our comparative results by northern blotting, and additionally determined absolute copy numbers of hsa-miR-155 in Raji and LCL 721 cells. As shown in Figure S1, quantification of northern blotting signals suggested 6-fold higher expression of hsa-miR-155 in the LCL cells, a value which is in perfect accord with our stem-loop PCR data. Absolute copy numbers of hsa-miR-155 per cell were determined to be in the range of 11,000–13,000 and 1,900–2,400 for LCL 721 and Raji cultures, respectively. Consequently, Raji cells express hsa-miR-155 at levels which are similar to those previously reported for kshv-miR-K12-11 in BCBL-1 cells. While we do not know the precise reasons for the difference between our results and those of Linnstaedt and colleagues, we hypothesize that one factor may be variability of latent gene expression patterns among Raji lines cultivated in our laboratories. As hsa-miR-155 is strongly induced by the viral LMP-1 gene product [50], [56], variable levels of LMP-1 expression may well be responsible for the observed differences.

Together, the experiments verify the functionality of generated γ-retroviruses. Importantly, the expression levels in transduced cell cultures were comparable to those observed in representative tumor cell lines of B-cell origin, indicating that retroviral transduction does not lead to gross overexpression of the respective miRNAs. We therefore proceeded to investigate the phenotypic consequences of constitutive miRNA expression *in vivo*.

### Ectopic hsa-miR-155 and kshv-miR-K12-11 expression results in increased pre-B-cell populations in the bone marrow of engrafted mice

To study phenotypic consequences of constitutive miRNA expression in the hematopoietic system, we transduced hematopietic progenitor cells/stem cells (HPC/HSC) isolated from bone marrow harvested from tibiae and femura of C57BL/6 mice, and transplanted the transduced cells into lethally irradiated recipient mice by tail vein injection. After 12 to 16 weeks, the mice were sacrificed and hematopoietic organs were analyzed. At the time of harvesting, the percentage of GFP-positive cells in the BM and splenic compartments of mice were generally between 0.5–30%. From this contingent, only mice exhibiting rates of at least 1% GFP positive cells were included in our study.

**Figure 2:**
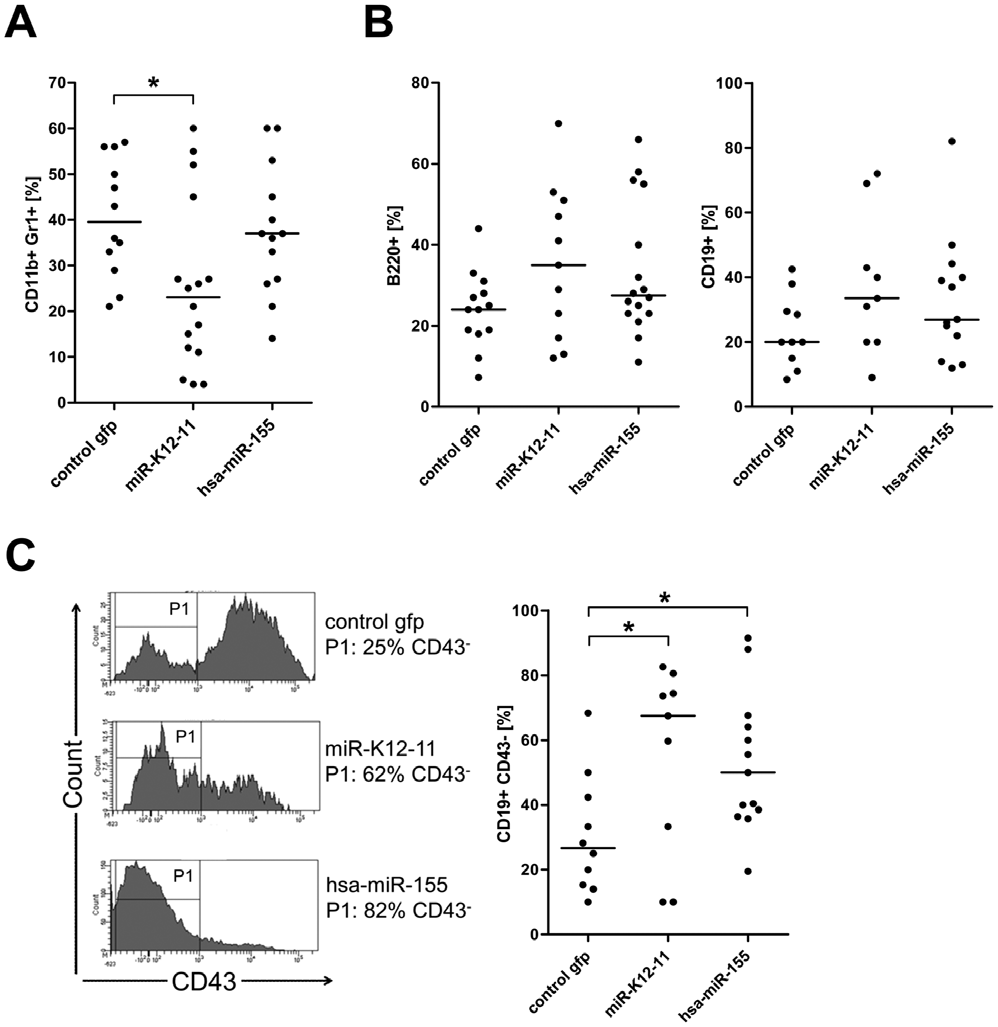
Increased fraction of pre B-cells in BM cells of miRNA-expressing mice. The immuno-phenotypic profile of BM cells harvested from tibiae and femora was characterized by flow cytometry. A) Decrease of myeloid population in miRNA-expressing mice. The graph represents flow cytometry results of GFP^+^ gated BM cells (total number of mice: control gfp: n = 12; kshv-miR-K12-11: n = 16; hsa-miR-155: n = 13). The result show a decrease of myeloid cell populations (CD11b^+^Gr1^+^) in the miRNA-expressing mice compared to control mice. B) Increase of B-cell population in the BM cells of miRNA-expressing mice. GFP^+^ gated BM cells from miRNA-expressing mice reveal a slight increase of B-lineage cells compared to GFP control mice (B220^+^ (left graph; (control gfp: n = 13; kshv-miR-K12-11: n = 11; hsa-miR-155: n = 16)) and CD19^+^ (right graph; (control gfp: n = 10; kshv-miR-K12-11: n = 9; hsa-miR-155: n = 13)). C) CD43 expression evaluated by flow cytometry on GFP^+^/CD19^+^ gated BM cells reveals a significant increase of the pre B-cell population (CD19^+^CD43^−^) in the BM of miRNA-expressing mice, indicating a shift toward the pre B-cell fraction. Left: histograms of one representative mouse each of miRNA-expressing and control mice. Gate P1 indicates the CD43^−^ population. Right: dot plots of all analyzed mice (control gfp: n = 10; kshv-miR-K12-11: n = 9; hsa-miR-155: n = 13).

We first performed a flow cytometry-based cell lineage analysis of GFP^+^ BM cells harvested from tibiae and femora using B-cell and myeloid markers. Interestingly, as shown in Figure 2A we observed a significant decrease of the myeloid cell population (CD11b+Gr1+) among the kshv-miR-K12-11 transduced cells relative to the control mice (22%; +/−18% and 40%; +/−12%, respectively). We also observed a weak overall reduction of myeloid cells in hsa-miR-155 mice (35%; +/−13%), however the extend of this reduction was not statistically significant. These observations suggested that differentiation towards the myeloid line is disfavored when kshv-miR-K12-11 is ectopically expressed, and that a similar trend might exist in hsa-miR-155 expressing cells.

**Figure 3:**
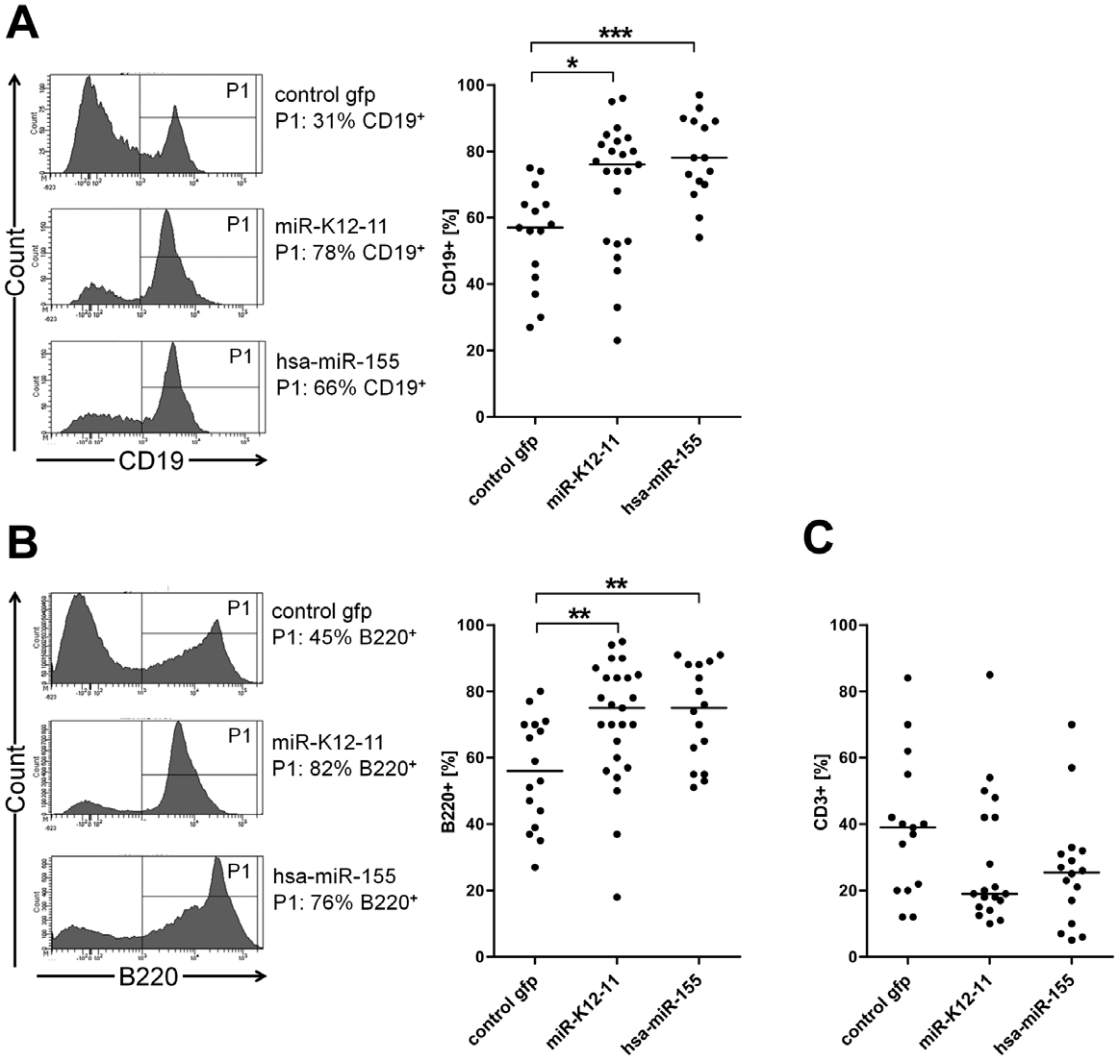
Expansion of B-cells in spleens of miRNA-expressing mice. Shown are analyses of GFP^+^ gated spleenocytes. A) Flow cytometry analysis reveals an expansion of B-cells in miRNA-expressing mice using the B-cell marker CD19. Left: histograms of one representative mouse each of each cohort. Gate P1 indicates CD19^+^ fraction. Right: dot plots of all analyzed mice (control gfp: n = 15; kshv-miR-K12-11: n = 23; hsa-miR-155: n = 15) reveal a significant shift towards the B-cell population miRNA-expressing mice. B) Flow cytometry analysis reveals an expansion of B-cells in miRNA-expressing mice using the B-cell marker B220. Left: histograms of one representative mouse per cohort. Gate P1 indicates the B220^+^ fraction. Right: dot plots of all analyzed mice (control gfp: n = 16; kshv-miR-K12-11: n = 25; hsa-miR-155: n = 16) reveal an expansion of B-cells among the GFP^+^ splenocytes. C) The number of T-cells is decreased in the miRNA-expressing mice cohorts, as indicated by flow cytometry analysis of the T-cell marker CD3 (control gfp: n = 15; kshv-miR-K12-11: n = 19; hsa-miR-155: n = 16).

We continued our cell lineage study by analyzing the B-cell population in GFP^+^ BM cells using B220 and CD19 antibodies (left and right panels, respectively, in figure 2 B). Although we did not detect statistically significant differences, we observed a slight but consistent shift towards the B-cell lineage in both miRNA-expressing mouse cohorts. This effect was more pronounced in the kshv-miR-K12-11 mice, in which the mean CD19^+^ population was 35% (+/−18%) of GFP^+^ gated BM cells compared to 20% (+/−9%) of control mice. The hsa-miR-155 mice showed 29% (+/−16%) CD19^+^ cells among the GFP^+^ BM cells. Although the effect was only moderate, the consistent increase of cells in the total B-cell population was in line with the notion that differentiation towards the myeloid lineage might be disfavored in kshv-miR-K12-11 (and potentially also hsa-miR-155) expressing cells, and suggested to us that more pronounced differences might exist among specific B-cell sub-populations. Indeed, when we analyzed the numbers of pro- and pre-B-cells, we detected a significant increase of the CD43^−^ pre B-cell population among the GFP^+^/CD19^+^ cells of both miRNA-expressing mouse-cohorts (Figure 2C). The left panels of Figure 3 C show exemplary histograms of one mouse from each mouse-cohort, whereas the results from all analyzed mice are shown in the graph to the right. As with the previously analyzed markers, the effects were more pronounced in the kshv-miR-K12-11 mice, which on average displayed 68% (+/−28%) pre B-cells, compared to 50% (+/−20%) and 28% (+/−18%) CD19^+^/CD43^−^ cells, respectively, for hsa-miR-155 and GFP-control mice. Our findings hence indicate that ectopic expression of both miRNAs in murine progenitor cells results in a shift from early pro B-cells (CD19^+^/CD43^+^, data not shown) towards the later stage pre B-cells (CD19^+^/CD43^−^) in the bone marrow. Given these observations, we continued our investigation in the splenic compartment.

### hsa-miR-155 and kshv-miR-K12-11 mediate B-cell expansion in the spleen

**Figure 4:**
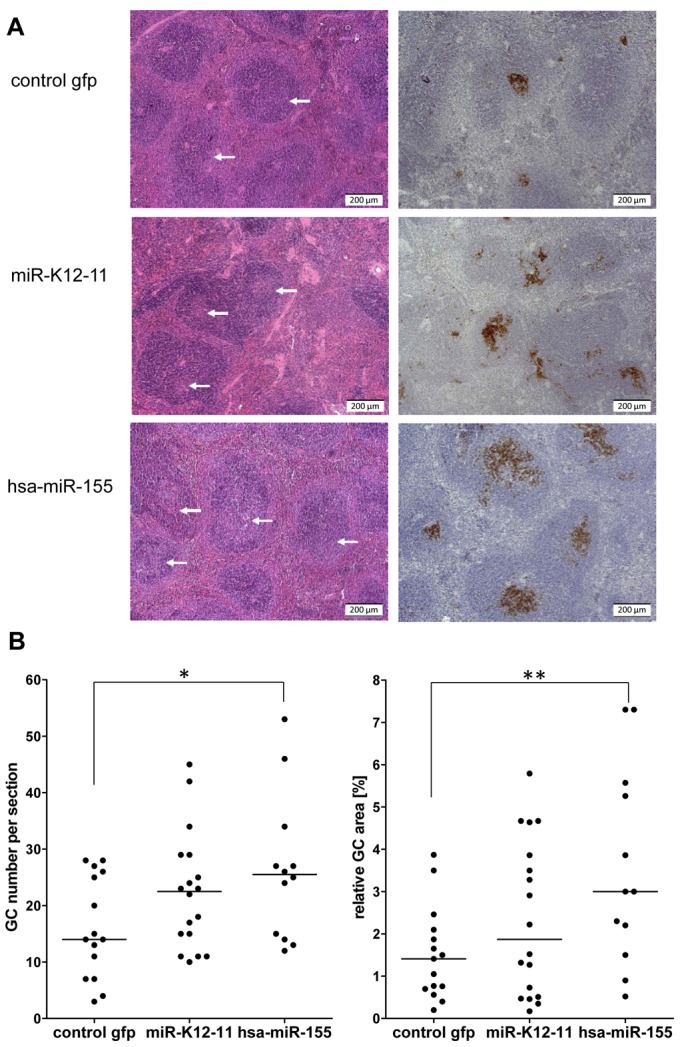
Enhanced GC development in miRNA-expressing mice. A) H&E staining and immunohistochemical detection if PNA indicate increased GC formation in miRNA-expressing mice. White arrows in H&E stained sections indicate the GC. PNA staining was used to determine the GC number and relative GC area per section. Shown are representative sections from one mouse of each cohort. B) Statistical analysis of the average number of GCs (left) and the relative total GC area (right) in the mouse cohorts. Enumeration of GCs and determination of total GC area was performed as described in the material and methods section.

To investigate whether the B-cell lineage in the spleen was affected by constitutive miRNA expression, we performed an immunophenotypic single cell analysis of splenocytes. As shown in Figure 3A, hsa-miR-155 as well as kshv-miR-K12-11 mice showed a significant increase of CD19^+^ B-cells among the GFP^+^ splenocytes. In the left panel of Figure 3A, we present FACS histograms to depict the shift to CD19^+^ B-cells in the miRNA-expressing mice relative to the control mice for one representative mouse of each cohort. As shown in the graphs to the right, the shift towards CD19^+^ B-cells in the miRNA-expressing mice was significant in the kshv-miR-K12-11 mouse cohort, with 72% (+/−19%) CD19^+^ cells, and highly significant in hsa-miR-155 mice with 75% (+/−12%) CD19^+^ cells, relative to 55% (+/−14%) CD19^+^ cells in the control mice. The shift towards the B-cell population was confirmed by detection of B220 as a second B-cell marker (Figure 3B). As for CD19, the percentage of B220^+^ splenocytes was significantly increased in both miRNA-expressing mice cohorts (75%+/−18% and +/−14% in the kshv-mIR-K12-11 and hsa-miR-155 cohorts, respectively, vs. 55% (+/−16%) in the control mice). To address the question whether the shift towards the B-cell fraction affects other hematopoietic cell lineages in the spleen, we analyzed the T-cell population by using a CD3 antibody. While we did not observe statistically significant differences, the average number of T-cells among GFP^+^ splenocytes was nevertheless decreased by approximately 1.8 and 2.2 fold in the hsa-miR-155a and the miR-K12-11-expressing mouse cohorts, respectively (Figure 4C).

Collectively, the above results suggest that constitutive expression of hsa-miR-155 or miR-K12-11 results in the accumulation of B-cells in the spleen, whereas the T-cell lineage may be disfavored when either miRNA is expressed. The accumulation of B-cells may be the result of differentiation or proliferation biases; alternatively, miRNA expression may mediate a selective pro-survival within the B-cell fraction.

### Enhanced GC formation in the miRNA-expressing mice

**Figure 5:**
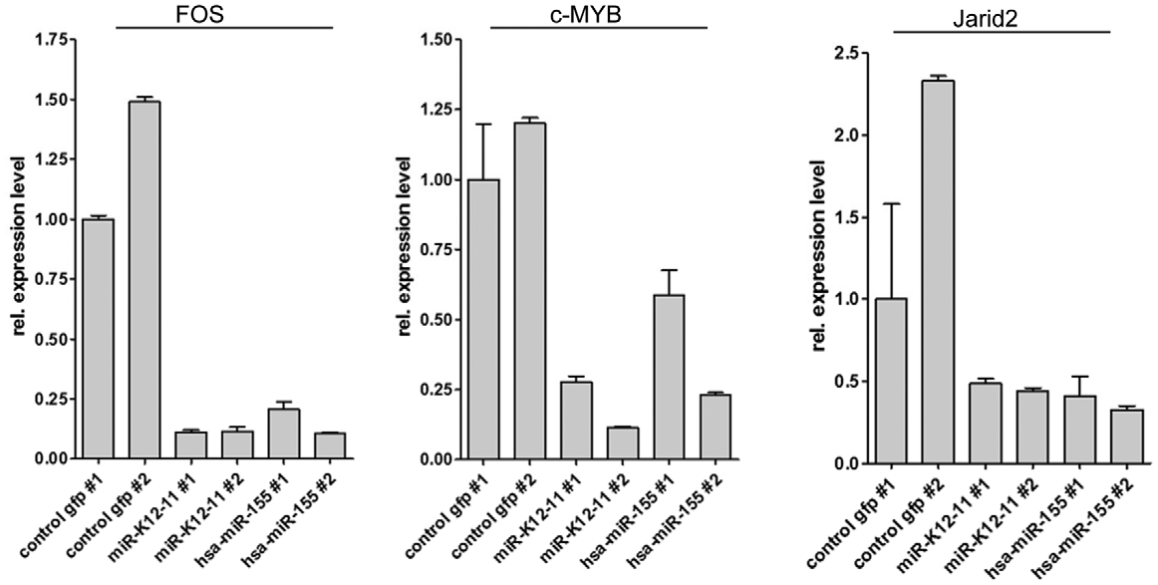
Decreased expression levels of miR-155 and kshv-miR-K12-11 target transcripts in miRNA expressing BM cells. Real-time RT PCR analysis was carried out using total RNA derived from flow sorted GFP^+^ BM cells from two mice of each mouse cohort. The mRNA levels for fos, c-myb and Jarid2 were normalized to endogenous level of actin, GAPDH and RPLP.

We next compared the efficiency of germinal center (GC) formation in the different mouse cohorts. For this purpose, we performed PNA staining to determine the total number of GCs, as well as the relative GC area, in paraffin-embedded sections of spleens from miRNA-expressing or control mice. Figure 4A shows H&E as well as PNA stainings from exemplary sections of each mouse cohort. As shown in Figure 4B (left panel), the statistical analysis revealed a significant increase in the numbers of GCs within the hsa-miR-155 mouse cohort (25; +/−12 GCs per section) when compared to the control mice (15; +/−9 GCs per section). The relative GC area (Figure 5B, right panel), calculated as the percentage of splenic area occupied by GCs across all sections, was likewise significantly enlarged in the hsa-miR-155 mice (3%; +/−2.2) relative to the controls (1.4%; +/−1). Kshv-miR-K12-11 mice displayed a similar trend towards increased GC numbers (22; +/−12 GCs per section) as well as slightly enlarged GC areas (1.9%; +/−1.5). Although the increase in GC numbers within the kshv-miR-K12-11 cohort did not pass the statistical significance threshold, the observed p-values were nevertheless low (0.073), suggesting that kshv-miR-K12-11, like hsa-miR-155, may enhance germinal center formation under conditions of constitutive expression.

### Jarid2 – a novel target of kshv-miR-K12-11

**Figure 6:**
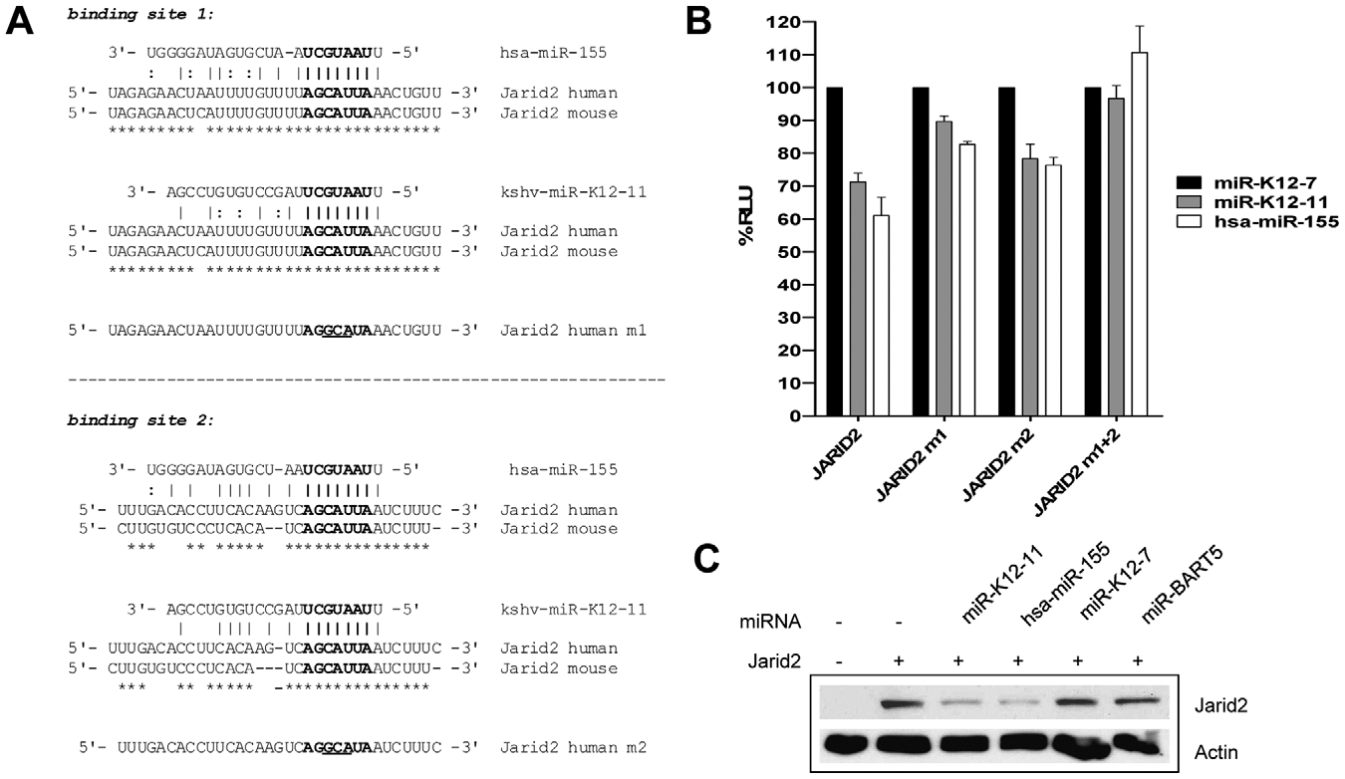
Jarid2 is targeted by hsa-miR-155 and kshv miR-K12-11. A) The 3′UTR of the prototypical human and mouse Jarid2 transcripts (GenBank accession numbers NM_004973 and NM_001205043, respectively) contains two seed match sites for kshv-miR-K12-11 and hsa-miR-155. Shown are alignments of the human and mouse 3′-UTR sequences, and their predicted pairing with hsa-miR-155 and kshv-miR-K12-11. Nucleotides that are conserved between mouse and human Jarid2 transcripts are marked by asterisks underneath the alignments. The sequences of mutated target sites are shown below the alignments in each panel. Mutated nucleotides are underlined. B) Luciferase reporter assay in 293T cells. Luciferase expression constructs harboring the wt Jarid2 3′UTR (JARID2), or variants in which target site 1 (JARID2 m1) or target site 2 (JARID2 m2) or both target sites (JARID2 m1+2) had been mutated were co-transfected with expression vectors for kshv-miR-K12-11, hsa-miR-155 or the irrelevant miRNA kshv-miR-K12-7. Results are shown normalized to kshv-miR-K12-7. C) Jarid2 protein levels are reduced when hsa-miR-155 or kshv-miR-K12-11 are expressed. 293T cells were co-transfected with a Jarid2 expression vector (GenBank accession BC046246) and expression constructs for kshv-miR-K12-11, hsa-miR-155 or the irrelevant control miRNAs kshv-miR-K12-7 or ebv-miR-BART5.

To evaluate the expression status of known or suspected hsa-miR-155 and kshv-miR-K12-11 targets in miRNA expressing cells, we performed RT-PCR to detect mRNA transcripts of the fos, c-myb and jarid2 genes Fos and c-myb were chosen because both genes are targeted by hsa-miR-155 and also have previously been reported to play important roles in B cell differentiation. Jarid2 is a member of polycomb repressor complexes, has been shown to be subject to miR-155 regulation in human and mouse cells [28], [39], [57], and was of particular interest since it had also emerged as a potential target of kshv-miR-K12-11 in one of our earlier screens (data not shown). As shown in Figure 5, in GFP^+^ BM cell isolated from two mice of each mouse cohort, the transcript levels of all three genes were consistently lower in miRNA-expressing compared to control mice. As kshv-miR-K12-11 has previously not been demonstrated to directly repress Jarid2, we next set out to validate Jarid2 as an authentic target of this viral microRNA. Additionally, we aimed to investigate repression of Jarid2 by miR-155 on the protein level, which had not been demonstrated before. As shown in Figure 6A, the Jarid2 3′-UTR contains 2 binding sites for hsa-miR-155 that are predicted to also represent functional target sites for kshv-miR-K12-11. With the exception of a single nucleotide exchange, target site 1 is perfectly conserved between mouse und humans (Figure 6A, upper panel). Target site 2 is more diverse by comparison, yet all nucleotides that are predicted to pair with miR-155 and kshv-miR-K12-11 in regions that are critical for target recognition [16] are conserved (Figure 6A, lower panel).

To verify that Jarid2 is a direct target gene of hsa-miR-155 as well as kshv-miR-K12-11, we first performed a luciferase reporter assay. The full-length 3′UTR of the prototypic Jarid2 transcript was cloned downstream of a luciferase gene in the pMIR-report vector, and was co-transfected with expression constructs for hsa-miR-155, miR-K12-11. An expression construct for an irrelevant miRNA (kshv-miR-K12-7) without canonical binding sites in the Jarid2 3′-UTR served as a negative and normalization control. As shown in the left columns of Figure 6B, consistent with previous reports [39], we observed repression of luciferase expression by approximately 40% when a hsa-miR-155 expression construct was co-transfected. kshv-miR-K12-11 was able to mediate repression to a similar extend (approx. 30% reduction). To ensure that repression was mediated by the predicted target sites, both sites were mutated individually as well as in the context of a double mutant (see Figure 5A for mutated sequences), and the luciferase assays were repeated. As shown in the rightward columns of Figure 6B, we found each site to contribute roughly 50% to the observed repression by hsa-miR-155 as well as kshv-miR-K12-11. Only the combined mutation of both sites restored luciferase expression to the same levels observed with the negative control. To confirm that expression of hsa-miR-155 or kshv-miR-K12-11 also leads to repression of protein expression from an authentic Jarid2-encoding mRNA transcript, we made use of an IMAGE Consortium Clone that expresses a shorter Jarid2 transcript variant containing target site 1 of the prototypical 3′-UTR (GenBank accession BC046246). The Jarid2 expression construct was introduced into 293T cells, along with expression construct for hsa-miR-155 or miR-K12-11. An empty vector and expression constructs for miR-K12-7 as well as a second irrelevant control, the Epstein Barr-Virus encoded ebv-miR-BART5, were used as negative controls. As shown in Figure 6C, expression of hsa-miR-155 as well as kshv-miR-K12-11, but not empty vector or the control miRNAs lead to a marked repression of Jarid2 protein levels.

Collectively, the above data confirm Jarid2 as a shared target of hsa-miR-155 and kshv-miR-K12-11 which, consistent with its downregulation in hsa-miR-155 and kshv-miR-K12-11 expressing BM cells, may be one of the factors contributing to the observed phenotypes *in vivo*.

## Discussion

About every tenth viral miRNA shares its seed sequence with a miRNA encoded by its host, a number which is approximately twice as high as would be expected to result from mere chance [20]. Although this observation suggests that a significant number of viral miRNAs have evolved to mimic host miRNAs, demonstration of common phenotypic consequences of miRNA expression, preferentially in *in vivo* model systems, is highly desirable. At the time when this study was initiated, strong experimental and circumstantial evidence had suggested that seed sharing between hsa-miR-155 and kshv-miR-K12-11 represents a case of high biological significance, yet *in vivo* models were still lacking. We therefore set out to study the impact of constitutive hsa-miR-155 and kshv-miR-K12-11 expression on hematopoietic stem cells in C57BL/6 mice.

Our findings indicate that forced expression of kshv-miR-K12-11 indeed re-capitulates important aspects of hsa-miR-155 expression. Firstly, in both miRNA-expressing mouse cohorts, we detected significantly increased frequencies of CD19^+^/CD43^−^ pre B-cells, but not an increase of the immature and ‘transient mature’ B220^+^IgM^+^ cell [58] population (data not shown). These data are in line with observation made by Croce and colleagues in an Eμ-mmu-miR155 transgenic mouse model [32] and suggest that ectopic expression of host as well as viral miRNAs increase the survival and/or proliferation rates of early pre B-cells. However, in striking contrast to this earlier study, we saw no evidence of pre B-cell leukemia, most likely reflecting different expression levels of miR-155, or the fact that not all pre B-cells express miR-155 in our system. Secondly, we also found expansion of B-cell pools in the spleen, a phenotype which is probably also the consequence of enhanced B-cell proliferation and/or survival. At least in our system, we consider minimally induction of pro-survival pathways to be likely, a scenario which is compatible with the observation of moderate underrepresentation of T-cells in the spleen and the fact that no significant changes in the total number of B- and T-cells were observed in the BM (data not shown). This scenario is furthermore supported by several studies that have reported induction of pro-survival pathways upon expression of miR-155 [59], [60]. Jarid2 may be one of the (likely multiple) factors that, when repressed, contributes to increased B-cell survival [39].

The finding that hsa-miR-155 as well as kshv-miR-K12-11 support B-cell expansion in the spleen is in accord with another study that was published while our work was in progress [42]. Whereas we additionally found an overrepresentation of B-cells specifically within the CD19^+^/CD43^−^ B-cell population of the BM, significant imbalances among BM cells were not apparent in the study by Boss et al. This may be explained simply by the fact that the specific CD19^+^/CD43^−^ B-cell population was not analyzed by Boss and colleagues. On the level of the total CD19^+^ B-cell population, however, both studies observed only a slight, statistically non-significant increase within the BM. Although both studies thus come to the same major conclusions, some noteworthy differences exist. Conceptually, the most important difference is that, whereas our study was performed in a murine background, the study by Boss and colleagues expressed the miRNAs in human hematopoietic progenitors, followed by immune reconstitution of NOD/LtSz-scid IL2Rγ null mice. Given that KSHV is a human pathogen, the use of a humanized mouse model at first would seem to be a superior system. Indeed, our system would be entirely unsuitable to study a viral miRNA that is not a putative mimic of a conserved host miRNA. This is because only such a mimic is predicted to have access to existing, evolutionary conserved networks of 3′-UTR target sites. In contrast, the unique target sites of other viral miRNAs are much less likely to be conserved across species. By the same token, however, for a viral miRNA that shares its seed with a conserved host miRNA, our system should preferentially conserve those shared targets that are engaged via the identical seed sequence, but not those that are targeted via unique sequences (e.g., the divergent 3′-terminus of kshv-miR-K12-11). Thus, we consider our system to provide a much more stringent environment to test the hypothesis that seed sharing is sufficient for a viral miRNA to phenocopy a host miRNA, and to therefore represent an ideal complement to other model systems such as the humanized mouse model employed by Boss and colleagues [42].

A second important difference is that our system allowed us the investigation of GCs, which are absent from HPC-reconstituted Nod/SCID mice. Interestingly, we observed increased GC numbers as well as enlarged GC areas in hsa-miR-155 and kshv-miR-K12-11 mice. A recent study by Thai and colleagues demonstrated that miR-155 influences GC reactions by controlling cytokine production [25], and we suspect that similar mechanisms may be responsible for the observations we made in the hsa-miR-155 and kshv-miR-K12-11 mouse cohorts. A caveat is that, in contrast to hsa-miR-155, the increase in GC number/area was not statistically significant for kshv-miR-K12-11 (however, the p-value for increased GC numbers was 0.073 and thus barely missed the significance mark). At present, we do not know why the kshv-miR-K12-11 mouse cohort exhibited greater variability. One possibility is that this observation simply reflects differences in the absolute numbers of human and viral miRNAs. In this context, it should be pointed out that, while we established that both miRNAs were expressed at appropriate and overall similar levels when compared to reference samples in *in vitro* transduced 3T3 cells (see Figure 1), due to limited amounts of RNA recovered from GFP-sorted cells we were not able to directly determine miRNA expression levels *in vivo*. Thus, we cannot fully exclude the possibility that, e.g. as a result of selectional processes, differences in the total abundance of kshv-miR-K12-11 and hsa-miR-155 may have contributed to some of the variability seen in our system.

Taken together, the observation of similar phenotypes in hsa-miR-155 and kshv-miR-K12-11 expressing mice strongly suggests that seed sharing is indeed sufficient to mimic hsa-miR-155 functions. If so, however, what is the physiological role of kshv-miR-K12-11 during the viral lifecycle? It would seem likely that the viral miRNA has evolved to allow KSHV to modulate B-cell development, rather than inducing hyperproliferation per se. In this context, it is noteworthy that neither our study nor that by Boss and colleagues, two studies that expressed the viral miRNA at near-physiological levels, observed the development of frank B-cell lymphoma. In analogy to models that have been put forth for EBV [61], we therefore favor a scenario in which kshv-miR-K12-11has primarily evolved to supplant activation signals that permit latently infected cells to transit germinal centers and differentiate into long lived memory cells (the likely long-term reservoir of persistent gammaherpesvirus infections). In EBV, such signals are provided by a number of viral factors that manipulate cellular signaling pathways, including the membrane proteins LMP-2A and LMP-1 (see [62]–[64] and references therein). The fact that EBV does not encode its own miR-155 mimic, but instead induces cellular miR-155 via LMP-1 [50], [65] supports the notion that kshv-miR-K12-11may play an important role during this process.

A number of miR-155 targets with important roles in B-cell differentiation, including Bach1, fos, c-myb, Pu.1 and C/EBPβ have been identified in the past [21], [24], [27], [28], [39], [42], [58], [66]. We have confirmed downregulation of two of the above genes (c-myb and fos) as well as of Jarid2 in kshv-miR-K12-11and hsa-miR-155 expressing BM cells, an observation which supports the idea that miR-155 may contribute to the silencing of these targets during B-cell development (it should be pointed out, however, that the marked repression very likely also involves other mechanisms that may directly act on the transcriptional level). Additionally, we validate Jarid2 as a novel target of kshv-miR-K12, and for the first time demonstrate that hsa-miR-155 as well as kshv-miR-K12-11can efficiently suppresses protein expression from an authentic Jarid2-encoding transcript.

C-myb and fos are cellular proto-oncogenes with important functions during hematopoiesis [67]–[71]. C-myb is of particular importance during early B-cell development and the pro B- to pre B-cell transition [68], [71]. C-myb was recently shown to be subject to regulation by miR-155 via two target sites in its 3′-UTR [72], and was earlier suggested as a target of kshv-miR-K12-11 due to its downregulation in BJAB-cells that express the viral miRNA [24]. Likewise, fos has been shown before to be a direct target of both miR-155 and kshv-miR-K12-11 [19], [24]. Regarding Jarid2, three recent studies showed that miRNA-155 decreases Jarid2 mRNA levels in human and mouse cells [28], [39], [57], and one of these studies additionally demonstrated repression of heterologous luciferase reporters bearing the Jarid2 3′-UTR by miR-155 [39]. Jarid2 is a co-factor of PRC2, a multi-component histone methyltransferase (HMT) complex with important functions in differentiation and cancer [73], [74]. PRC2 complexes mediate di- and trimethylation of histone H3 at lysine 27 (H3K27me2 and -me3, respectively). The H3K27me3 mark results in transcriptional repression, which is presumably maintained by binding of the polycomb repressor complex 1 (PRC1). Besides of its key role in the suppression of differentiation genes in embryonic stem cells, PRC2 has been found to restrict the activity of hematopoietic stem and precursor cells [75]–[77]. Additionally, deregulation of PRC2's methyltransferase activity is a frequent feature of several cancers, including carcinoma, neuroblastoma and hematologic malignancies (for recent reviews, see [78]–[80]). Interestingly, we have previously found that PRC2 is also of fundamental importance for the establishment of latent KSHV infection: Soon after entry into the host cell nucleus, the viral genome becomes subject to abundant H3K27 tri-methylation. This mark precedes the emergence of DNA methylation patterns, and confers a rapid repression of viral genes that otherwise would induce the lytic cycle [81].

Jarid2 has been shown to exert negative regulation on the activity of PRC2 complexes [82]–[84], and to contribute to the transcriptional activation of a subset of genes that are bound by PRC2 [82]. While it has also been reported that Jarid2 can positively influence repressive PRC2 activity [85], the functional basis for this conflicting result is unknown; it is possible that the outcome of regulation may depend on additional factors such as chromatin context or specific composition of PRC2 complexes (see [41] for a review of this issue). Interestingly, a number of recent reports suggest that Jarid2 may act as a tumor suppressor in hematopoietic malignancies [86]–[88], a function which would be compatible with its negative influence on cell growth [88] and cell survival [39]. Given the above, it is conceivable to assume that repression of Jarid2 via kshv-miR-K12-11may play an important role during latent infection of B-cells by KSHV. Indeed, a recent study that used PAR-CLIP technology to identify putative targets of KSHV miRNAs in PEL cell lines recovered one of the Jarid2 target sites validated in our study, indicating that Jarid2 is also targeted by kshv-miR-K12-11 in KSHV-associated tumors [19]. Interestingly, Beemon and colleagues reported that repressed Jarid2 levels are associated with increased B-cell survival [39], which potentially could contribute to the phenotypic observations made in our mouse model.

Taken together, our data strongly support previous studies that have suggested that kshv-miR-K12-11has evolved to mimic host miR-155. While the primary function of kshv-miR-K12-11 may be to modulate B-cell development, it is easy to see how these manipulations may pre-dispose the host cell to malignant transformation, especially in the context of the expression of other latency genes such as LANA, v-Flip or v-cyclin. It is well possible that co-expression of these factors, or even expression of the viral miRNA alone at levels that are higher than those which were used here, would lead to the development of proliferative disease such as has been observed in other mouse models of forced miR-155 expression.

## Supporting Information

Figure S1 **Quantification of hsa-miR-155 expression levels in EBV-positive B cell lines.** A) Determination of absolute copy numbers of hsa-miR-155 per cell in Raji and LCL 721 cultures. Copy numbers were determined by real-time stem-loop PCR, using a standard curve generated with synthetic miRNAs as described in Methods S1. B) Detection of hsa-miR-155 in Raji, LCL 721 and Jijoye cells by northern blotting (left panel). Signal intensities of bands corresponding to the mature miRNAs (23 nt) were determined using a phosphoimager (see Methods S1 for details), and are indicated underneath the blot as percentage values relative to Raji. For comparison, kshv-miR-K12-11 was detected in the same samples in the left panel. To ensure equal loading in each lane, ethidium bromide staining of the gels prior to transfer was used to detect ribosomal RNA moieties (shown above the blots in each panel).
(TIF)

Methods S1 (PDF)
