# Supplementary figures and images for: A microRNA Encoded by Kaposi Sarcoma-Associated Herpesvirus Promotes B-Cell Expansion In Vivo
Source: PLoS One. 2012 Nov 20;7(11):e49435. doi: 10.1371/journal.pone.0049435 (PMC3502504; doi:10.1371/journal.pone.0049435)

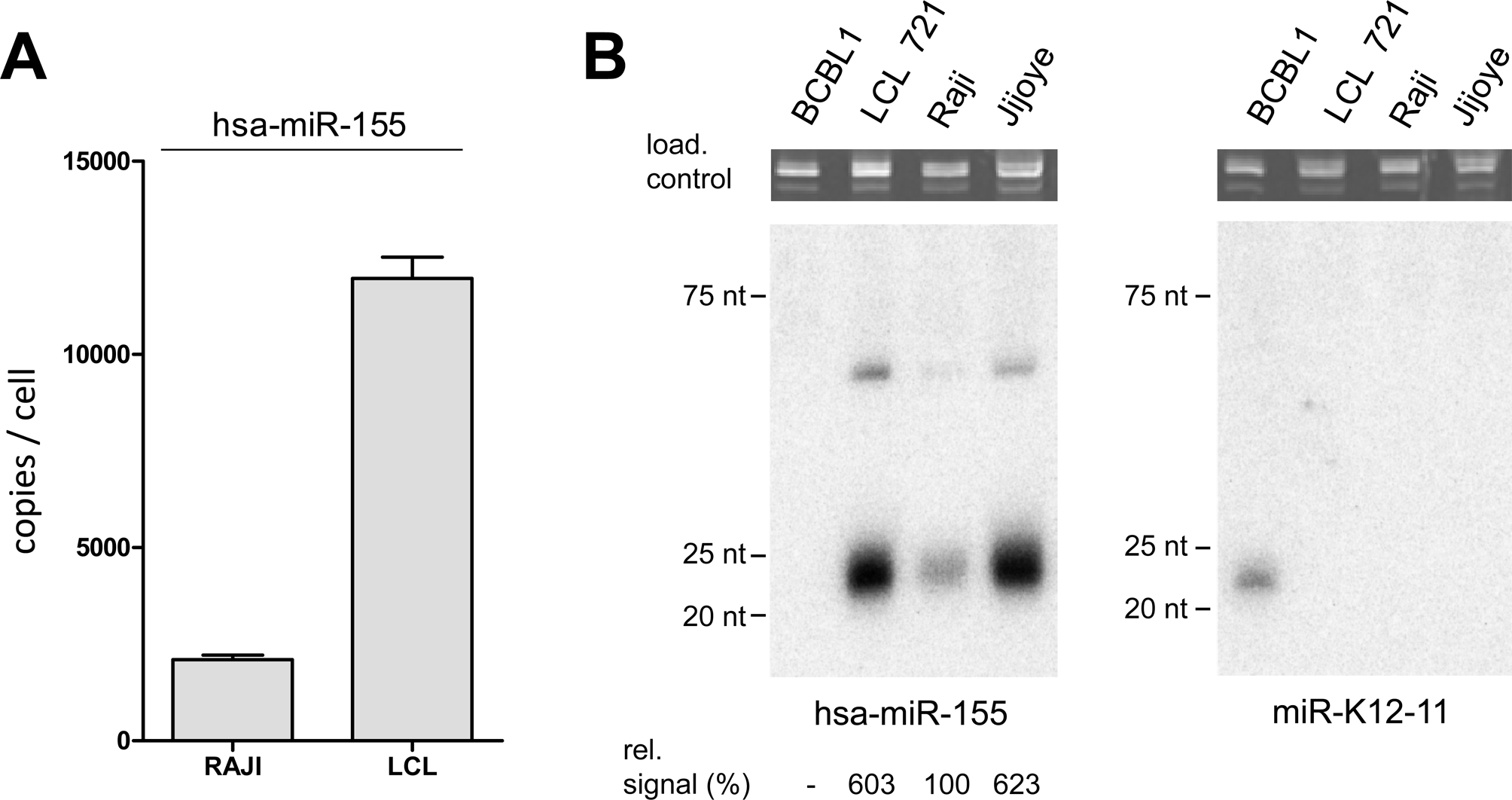

Supplement: Figure S1 — Quantification of hsa-miR-155 expression levels in EBV-positive B cell lines. A) Determination of absolute copy numbers of hsa-miR-155 per cell in Raji and LCL 721 cultures. Copy numbers were determined by real-time stem-loop PCR, using a standard curve generated with synthetic miRNAs as described in Methods S1. B) Detection of hsa-miR-155 in Raji, LCL 721 and Jijoye cells by northern blotting (left panel). Signal intensities of bands corresponding to the mature miRNAs (23 nt) were determined using a phosphoimager (see Methods S1 for details), and are indicated underneath the blot as percentage values relative to Raji. For comparison, kshv-miR-K12-11 was detected in the same samples in the left panel. To ensure equal loading in each lane, ethidium bromide staining of the gels prior to transfer was used to detect ribosomal RNA moieties (shown above the blots in each panel). (TIF) [file pone.0049435.s001.tif]
