# Supplementary material for: A microRNA Encoded by Kaposi Sarcoma-Associated Herpesvirus Promotes B-Cell Expansion In Vivo
Source: PLoS One. 2012 Nov 20;7(11):e49435. doi: 10.1371/journal.pone.0049435 (PMC3502504; doi:10.1371/journal.pone.0049435)
Supplement: Methods S1 — (PDF) [file pone.0049435.s002.pdf]

Supporting Methods

Northern Blot Analysis

Small RNA analysis by northern blotting was performed as previously described (Walz et al., 2010). Briefly, 15% SDS gels containing 8 M urea were pre-run for 1 h at 45 mA. 14 µg of total RNA were mixed with an equal volume of formamide containing loading dye and heat denatured for 2 min at 70 °C. RNA was then separated for 60 min at 35 mA. The gels were stained in a 4 µg/ml ethidium bromide solution and evaluated under UV light for RNA integrity and equal loading. Gels were then washed in 1x TBE, and RNA was transferred to a Zeta-Probe Membrane using semi-dry-blotting in 1x TBE for 2 h at 2.08 mA/cm^2. The membrane was washed in 1x TBE, dried and cross-linked with UV light and pre-hybridized for 1 h at 37 °C in hybridization buffer (ExpressHyb, BD Biosciences) in a rotating hybridization oven. 20 pmoles of oligonucleotide probes specific for hsa-miR-155 (5'- ACC CCT ATC ACG ATT AGC ATT AA -3') or miR-K12-11 (5'- TCG GAC ACA GGC TAA GCA TTA A -3') were mixed with 10U Polynucleotide kinase (USB), 5 µl of 10x PNK buffer, 25 µl γP32-dATP (SRP-501, Hartmann-Analytic) and DEPC H_2O to a final volume of 50 µl and labeled for 1 h at 37 °C. PNK was inactivated by heating the probes for 10 min at 65 °C, un-incorporated nucleotides were separated from the probe using G-25 Sephadex columns. Blots were hybridized overnight in ExpressHyb solution (BD Biosciences) according to the manufacturer's instructions. After washing, blots were exposed to a BAS-MP 2040P Imaging Plate (Fuji Photo Film Co., LTD.) for 24 h. The plate was scanned with a BAS-2500 scanner (Fujifilm), and Aida Image Analyzer software (raytest) was used to quantify the signal levels of individual bands.

Determination of absolute hsa-miR-155 copy numbers

For the determination of absolute copy numbers of hsa-miR-155 in the B cell lines RAJI and LCL 721, we generated standard curves using known quantities of a synthetic RNA molecule (Invitrogen) with the exact same sequence as hsa-miR-155 (5'- UUA AUG CUA AUC GUG AUA GGG GU -3'). A 100 µM solution of the synthetic miRNA was diluted to a concentration to $1x10^8$ copies per µl, followed by reverse transcription with SuperScript III (Invitrogen) and preparation of 10 fold serial dilutions ranging over 6 orders of magnitude. A standard curve was then generated using

real-time stem-loop RT PCR as described in the material & methods section of the main manuscript. To estimate absolute hsa-miR-155 copy numbers per cell, RNA was isolated from $5x10^6$ RAJI or LCL 721 cells and quantified with the Quant-iT™ RNA Assay Kit on a Qubit® fluorimeter (Invitrogen) to determine average RNA content per cell (RAJI: 15.42 pg/cell; LCL 721: 17.37 pg/cell). 100 ng of RNA was then subjected to real-time stem-loop RT PCR and absolute miRNA copy numbers per cell were calculated using the miRNA standard curve generated as described above.
